# Antibody drug separation using thermoresponsive anionic polymer brush modified beads with optimised electrostatic and hydrophobic interactions

**DOI:** 10.1038/s41598-020-68707-7

**Published:** 2020-07-27

**Authors:** Kenichi Nagase, Saki Ishii, Koji Ikeda, Sota Yamada, Daiju Ichikawa, Aya Mizutani Akimoto, Yutaka Hattori, Hideko Kanazawa

**Affiliations:** 1 Faculty of Pharmacy, Keio University, 1-5-30 Shibakoen, Tokyo, Minato 105-8512 Japan; 2 Department of Materials Engineering, School of Engineering, The University of Tokyo, 7-3-1 Hongo, Bunkyo, Tokyo 113-8656 Japan

**Keywords:** Biotechnology, Chemistry, Materials science, Nanoscience and technology

## Abstract

Antibody drugs play an important role in biopharmaceuticals, because of the specificity for target biomolecules and reduction of side effects. Thus, separation and analysis techniques for these antibody drugs have increased in importance. In the present study, we develop functional chromatography matrices for antibody drug separation and analysis. Three types of polymers, poly(*N*-isopropylacrylamide (NIPAAm)-*co*-2-acrylamido-2-methylpropanesulfonic acid (AMPS)-*co-N*-phenyl acrylamide (PhAAm)), P(NIPAAm-*co*-AMPS-*co-n*-butyl methacrylate (BMA)), and P(NIPAAm-*co*-AMPS-*co*-*tert*-butylacrylamide (tBAAm)), were modified on silica beads through atom transfer radical polymerisation. Rituximab elution profiles were observed using the prepared beads-packed column. Rituximab adsorption at high temperature and elution at low temperature from the column were observed, as a result of the temperature-modulated electrostatic and hydrophobic interactions. Using the column, rituximab purification from contaminants was performed simply by changing the temperature. Additionally, three types of antibody drugs were separated using the column through temperature-modulated hydrophobic and electrostatic interactions. These results demonstrate that the temperature-responsive column can be applied for the separation and analysis of biopharmaceuticals through a simple control of the column temperature.

## Introduction

Recently, biopharmaceuticals have become effective medical treatments for refractory disease^1^. Antibody drugs play an important role in biopharmaceuticals, because of the specificity for target biomolecules and reduction of side effects^1–3^. Thus, separation and analysis techniques for these antibody drugs have increased in importance. Various types of separation and analysis techniques have been developed. Affinity chromatography using protein A ligand is a powerful tool for this purpose, because of the effective affinity between protein A and antibodies^4,5^. However, antibody elution from the columns is performed using a lower pH aqueous solution. The elution process includes the possibility of losing the activity of the antibody drugs and contamination owing to the elution of protein A. Thus, an antibody drug separation column that uses different retention and elution mechanisms from protein A is demanded.

Temperature-responsive chromatography is one of the candidates as an antibody separation technique, because the chromatography system can modulate the retention and elution of analytes by changing the temperature^6–9^. The chromatography column uses a thermoresponsive polymer, poly(*N*-isopropylacrylamide) (PNIPAAm). This polymer exhibits temperature-dependent hydrophilic and hydrophobic changes across the lower critical solution temperature (LCST) of 32 °C, owing to hydration and dehydration^10^. This temperature responsive property has been widely used in biomedical fields^11–13^, such as drug and gene delivery systems^14,15^, biosensors and diagnostic devices^16–18^, and cell culture dishes for tissue engineering and regenerative medicine^19–23^. In the application of PNIPAAm to chromatography for temperature-responsive chromatography, PNIPAAm-modified beads are used as the chromatography column^24–27^. The chromatography column can modulate the retention of analytes by changing the column temperature, because the surface hydrophobicity of the beads is changed with the hydration and dehydration of the modified PNIPAAm, which leads to the modulation of the hydrophobic interaction with analytes.

Additionally, temperature-responsive ion exchange chromatography has been investigated for the separation and analysis of ionic biomolecules and pharmaceutical proteins through temperature-modulated electrostatic interaction with analytes^28–34^, because bioactive compounds and proteins have an ionic property and the use of electrostatic interactions is an effective approach for separation. Temperature-responsive ion exchange chromatography uses thermoresponsive ionic copolymer modified beads as the packing material. The electrostatic interaction can be modulated by changing the column temperature. The thermoresponsive ionic copolymer is prepared by copolymerisation with an ionic monomer and NIPAAm. Furthermore, a hydrophobic monomer is incorporated to adjust the lower critical solution temperature, because the ionic monomer provides hydrophilicity to the polymer, which leads to an increase of the LCST of the polymer^35–37^. The main driving force of temperature-responsive ion exchange chromatography is the electrostatic interaction. Thus, various types of thermoresponsive ionic polymers have been investigated as ligands in thermoresponsive ion exchange chromatography by changing the incorporated ionic monomer^28,29,38,39^. Using the silica beads modified with the thermoresponsive ionic polymer P(NIPAAm-*co*-acrylic acid (AAc)-*co-n*-butyl methacrylate (BMA)) as the packing material of the solid phase extraction column, the antibody drug rituximab was separated from a contaminant^40^. However, in this attempt, AAc was used as the ionic monomer. AAc has a carboxyl group, which is a weakly acidic group that leads to a weak electrostatic interaction with the targeted analytes. Thus, if a thermoresponsive polymer with a strongly acidic group is used in the packing material, a more effective antibody drug separation column would be developed.

Among the strongly acidic monomers, 2-acrylamido-2-methylpropanesulfonic acid (AMPS) has been shown to be an effective ionic co-monomer owing to its strong acidic property^39,41^. However, depending on the property of the analytes, a hydrophobic interaction with the analytes can also contribute to the retention of the analyte^37^. Thus, if the hydrophobic interaction in addition to the electrostatic interaction with analytes are optimised, the separation efficiency of the temperature-responsive ion exchange column will increase remarkably, which will lead to the development of an effective antibody drug separation column.

**Figure 1:**
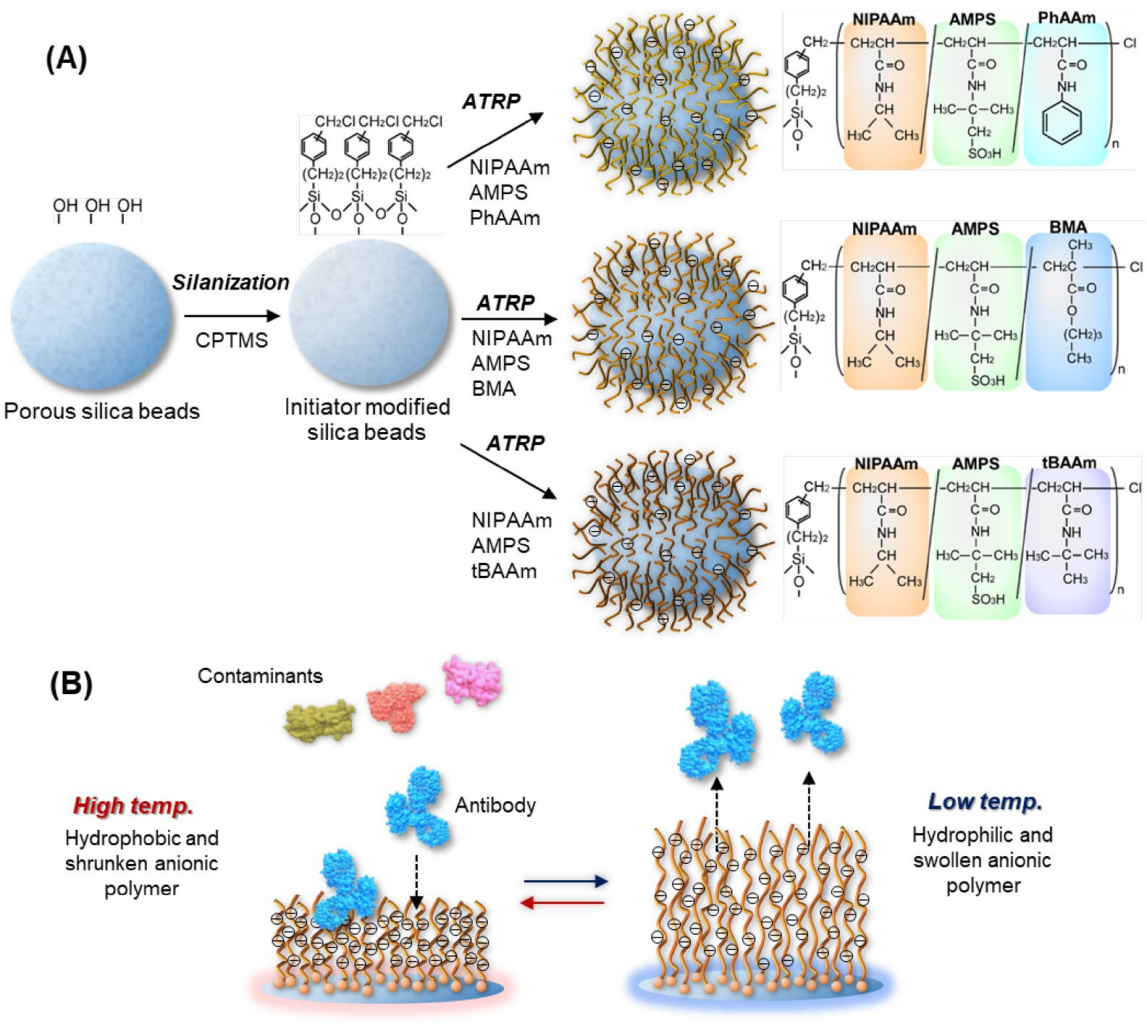
Schematic illustration of the preparation of thermoresponsive anionic copolymer brushes modified beads with various hydrophobic groups (**A**) and temperature-modulated antibody adsorption and desorption on the anionic polymer brush modified beads (**B**).

In the present study, to optimise the electrostatic and hydrophobic interactions with the antibody, we have prepared three types of thermoresponsive anionic polymer modified silica beads having the acidic AMPS monomer and three types of hydrophobic monomers, *tert*-butylacrylamide (tBAAm), *n*-butyl methacrylate (BMA), and *N*-phenylacrylamide (PhAAm), for the effective separation of an antibody (Fig. 1). Among the three polymers, P(NIPAAm-*co*-AMPS-*co*-PhAAm) and P(NIPAAm-*co*-AMPS-*co*-BMA) have not been investigated as a ligand for temperature-responsive ion exchange chromatography. Although P(NIPAAm-*co*-AMPS-*co*-tBAAm) has already been investigated as a temperature responsive chromatography material for the adsorption of lysozyme and α-chymotrypsinogen A, antibody separation has not been investigated using polymer modified beads^39^.

The electrostatic and hydrophobic interactions between the prepared beads and antibody drug are investigated by the elution behaviour of the antibody drug from the prepared beads-packed column. From the results, an effective temperature-modulated antibody drug separation column is developed. The developed column is able to separate the antibody without losing activity or using protein A ligand, which cause deactivation of the antibody and contamination of the products.

## Results and discussion

### Characterisation of the beads

**Figure 2:**
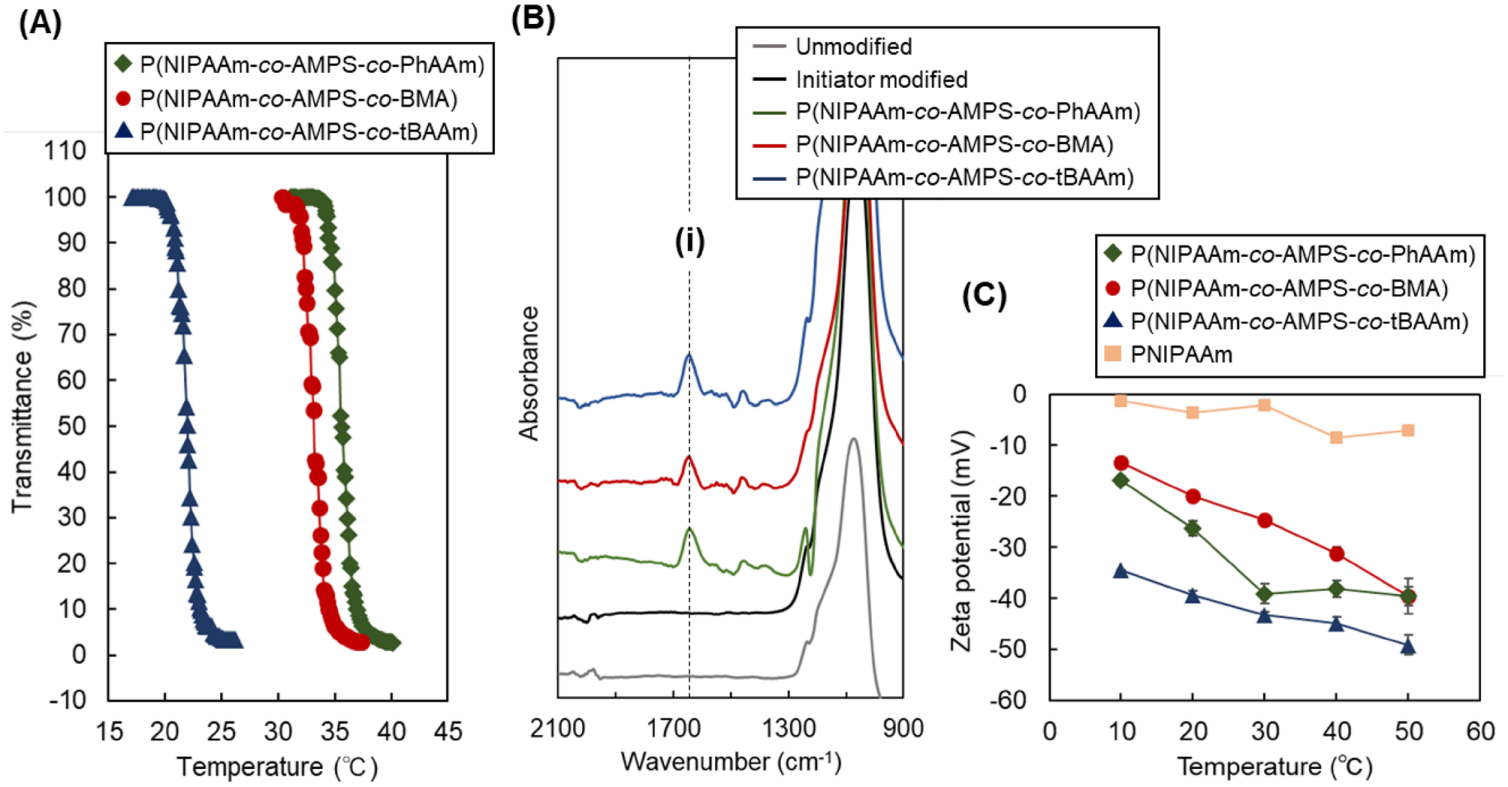
Characterisation of the prepared beads and polymer. (**A**) Phase transition behaviour of the temperature-responsive copolymer. Phosphate buffer solution (pH 7.0) is used as the solvent. (**B**) FTIR spectra of the prepared beads. The dashed line (i) represents the peak attributed to C=O bond. (**C**) Zeta potential of the prepared beads.

Unbound thermoresponsive polymers with various hydrophobic co-monomers and the same composition of polymers for modifying the silica beads were prepared to investigate the phase transition property (Fig. 2A). The monomer compositions of the polymers are summarized in Table 1. The AMPS composition was set at 5 mol%, which is the maximum amount incorporated into the thermoresponsive polymer^39^. The compositions of BMA and tBAAm were set at 5 mol% and 20 mol%, respectively, to modulate the proper phase transition temperature according to the previous reports^39,42^. The composition of PhAAm was not determined, since the copolymer was not investigated. Thus, we set the composition of PhAAm to 1 mol% in the first preparation. The hydrophobicity, log*P*, of the hydrophobic monomers, PhAAm, BMA, and tBAAm, were calculated using Crippen’s fragmentation^43^, and these values were 1.63 for PhAAm, 2.23 for BMA, and 0.84 for tBAAm (Supplementary Table S1). All polymers exhibited a phase transition from 10 to 50 °C, which indicated that the polymer property can be modulated to interact with analytes by changing the temperature within this temperature range. The phase transition temperature of P(NIPAAm-*co*-AMPS-*co*-PhAAm) was relatively high. Thus, we prepared P(NIPAAm-*co*-AMPS-*co*-PhAAm) including 2 mol% and 3 mol% of PhAAm to lower the phase transition temperature. However, these polymers were insoluble in a phosphate buffer solution even at low temperature (Supplementary Fig. S1). This was because the relatively strong hydrophobicity of PhAAm (log*P* = 1.63) prevented the hydration of the polymer. The results indicated that the composition of PhAAm in the copolymer could not be increased to above 1 mol%.

**Table 1:** Characterisation of temperature-responsive anionic polymer brush modified beads.

Sample	Monomer feed ratio (mol%)			Elemental composition (%)		%C_(calcd)_	Immobilised initiator (µmol/m^2^)	Grafted polymer (mg/m^2^)
	NIPAAm	AMPS	Hydrophobic monomer	C	N			
Unmodified silica beads				0.54 ± 0.04	0.33 ± 0.09			
Initiator-modified beads				4.16 ± 0.01	1.91 ± 1.26	0.595	4.02	
P(NIPAAm-*co*-AMPS-*co*-PhAAm)-modified beads	94	5	1	16.27 ± 0.09	2.82 ± 0.05	0.626		2.47
P(NIPAAm-*co*-AMPS-*co*-BMA)-modified beads	90	5	5	16.28 ± 0.06	2.49 ± 0.01	0.627		2.47
P(NIPAAm-*co*-AMPS-*co*-tBAAm)-modified beads	75	5	20	13.81 ± 1.02	3.04 ± 0.02	0.630		1.83

Elemental composition was determined by CHN elemental analysis. %C (calcd) was calculated by the ratio of the molecular weight of carbon in each monomer to the total molecular weight of each monomer. Amounts of initiator and polymer on silica beads were estimated using the carbon composition.

Atom transfer radical polymerisation (ATRP)-initiator-modified silica beads were prepared by a silanization reaction of [(chloromethyl)phenylethyl] trimethoxysilane (CPTMS), and the polymer was modified on silica beads through ATRP (Fig. 1A). The prepared initiator- and polymer-modified beads were characterised by CHN elemental analysis and the amounts of initiator and polymers were obtained (Table 1). The carbon composition increased after each reaction step, such as initiator modification and polymer modification, which indicated that each reaction step successfully proceeded. The initiator amount on silica beads was 4.02 µmol/m^2^, which was almost the same value as for silane modification on silica beads^44^. The amount of polymer modification ranged from 1.83 to 2.47 mg/m^2^, which indicated that a relatively large amount of polymer was in the form of successfully-modified silica beads, which contributed to a densely packed polymer brush conformation^44,45^. P(NIPAAm-*co*-AMPS-*co*-tBAAm)-modified beads exhibited a relatively small amount of polymer modification compared with those of P(NIPAAm-*co*-AMPS-*co*-PhAAm)-modified beads and P(NIPAAm-*co*-AMPS-*co*-BMA)-modified beads. This was proposed to arise from the reactivity of each hydrophobic monomer and the difference in monomer composition. Acrylamide monomers, such as PhAAm and tBAAm, have a low reactivity in the ATRP reaction compared with methacrylate monomers, such as BMA. Additionally, the reactivity of tBAAm would be low compared with NIPAAm, because of the larger monomer size of tBAAm compared with NIPAAm. Furthermore, the tBAAm composition was 20 mol%, which was quite high. Thus, the amount of P(NIPAAm-*co*-AMPS-*co*-tBAAm)-modified beads was relatively small compared with the other beads.

Previous reports indicated that the chain length of the polymer on silica beads affects the separation efficiency of analytes^24,42,44,46^. Thus, to investigate the difference in the molecular weight of the grafted thermoresponsive polymers on beads, unbound thermoresponsive polymers were prepared by the same polymerization condition, except that an unbound ATRP initiator, α-chloro-*p*-xylene, was used in place of the ATRP-initiator, CPTMS, modified beads. The molecular weight of the synthesized polymer was measured by a gel permeation chromatography (GPC) system. There was no significant difference in the molecular weight of the three polymers, P(NIPAAm-*co*-AMPS-*co*-PhAAm), P(NIPAAm-*co*-AMPS-*co*-BMA), and P(NIPAAm-*co*-AMPS-*co*-tBAAm) (Supplementary Table S2).

The prepared beads for each reaction step were characterised by Fourier-transform infrared spectroscopy (FTIR) measurements (Fig. 2B). An additional peak was observed at 1,650 cm^−1^ for the three types of polymer-modified beads, while the unmodified silica beads and initiator-modified beads did not exhibit this peak. The peak was attributed to the C=O bond of the amide group in the polymers. This also indicated that polymer modification on silica beads was successfully performed through the ATRP procedure in this study.

The morphology of the beads was observed by field-emission scanning electron microscopy (FE-SEM; Supplementary Fig. S2). All of the beads maintained spherical shapes, which indicated that the salinization reaction and polymer modification through ATRP does not deform silica-based materials. Additionally, the aggregation of the beads and increase in the diameter of the beads were not observed. The results indicated that the polymerisation reaction during polymer modification was controlled.

The surface area of the beads was measured by nitrogen adsorption (Supplementary Table S3). By comparing the surface area and peak pore diameter with those of unmodified beads (surface area: 100 m^2^/g, peak pore diameter: 30 nm), the values were decreased after the thermoresponsive anionic polymer modification. This is because the thermoresponsive polymer was modified inside the pore of the beads through the ATRP reaction, and the polymer filled the pore, which led to a reduction of the surface area.

To investigate the electrostatic property of the prepared beads, the zeta potential of the beads was measured (Fig. 2C). The zeta potential of the beads decreased with increasing temperature. This result differed from that of the thermoresponsive ionic copolymer modified beads^29^. Previous results indicated that the ionic property of the thermoresponsive ionic polymer decreased with increasing temperature, because the dissociation and protonation of the introduced ionic group was suppressed by the increased hydrophobicity of the thermoresponsive polymer adjacent to an ionic group, which induced dehydration of the thermoresponsive polymer^36,47^. However, the zeta potential of the prepared polymer-modified beads exhibited an opposite tendency to the previous reports. This was attributed to the strong anionic property of the AMPS in the thermoresponsive polymer. In the case of the previous reports, the ionic group in the thermoresponsive polymers was a weak acid or base, such as a carboxyl group or dimethyl amino group. These ionic groups tend to associate or become deprotonated by changing the hydrophobic property in the vicinity of the ionic group. However, the sulfonic acid group of AMPS always dissociates independent of the hydrophobicity in the vicinity of the sulfonic acid group, because the sulfonic acid group is a strongly acidic group. In addition, the thermoresponsive brush shrunk with increasing temperature, which led to an increase in the apparent charge density of the brush surfaces on silica beads. Thus, the zeta potential of the prepared beads decreased with increasing temperature.

To compare the zeta potential of the non-anionic thermoresponsive polymer modified beads, PNIPAAm-modified silica beads were prepared by the same procedure as that of thermoresponsive anionic polymer modified beads except that all the monomer was changed to NIPAAm. The zeta potential of the PNIPAAm beads slightly decreased with increasing temperature (Fig. 2C). A previous report indicated that the zeta potential of the PNIPAAm-modified surface decreases with increasing temperature, which was attributed to the collapse of the surface electrostatic double layer^29^. Thus, in a similar way, the zeta potential of the PNIPAAm brush modified beads slightly decreased with increasing temperature. In addition, the base silica beads have an anionic property, and this property may be concealed or exposed by the grafted PNIPAAm. At low temperature, the modified PNIPAAm on silica beads became swollen and concealed the surface anionic property of base silica beads. In contrast, at high temperature, the modified PNIPAAm on silica beads shrunk and exposed the anionic property of the base silica beads. These factors would lead to a slight decrease in the zeta potential of the PNIPAAm-modified beads.

The zeta potential change with temperature exhibited a broader change (Fig. 2C), while the phase transition behaviour of the polymer exhibited a sharp change with changing the temperature (Fig. 2A). This was attributed to the increased charge density induced by the shrinking of the thermoresponsive anionic polymers by increasing the temperature arising from the difference in the polymer graft configuration^44,48,49^. The unbound thermoresponsive polymer, dissolved in solution, exhibited a sharp turbidity change, because the polymer tended to change its structure by swelling and shrinking. In contrast, the modified polymer on the silica beads, especially the densely packed polymer brush structure, had its mobility restricted and had difficulty in changing its structure^44^. Thus, the zeta potential change with temperature exhibited a broad change with temperature.

The change in the diameter of the beads with changing temperature was investigated by dynamic light scattering (Supplementary Fig. S3). The diameter of the beads slightly decreased with increasing temperature, probably because the modified thermoresponsive anionic polymers shrunk with increasing temperature.

### Electrostatic and hydrophobic interactions between polymer and analytes

**Figure 3:**
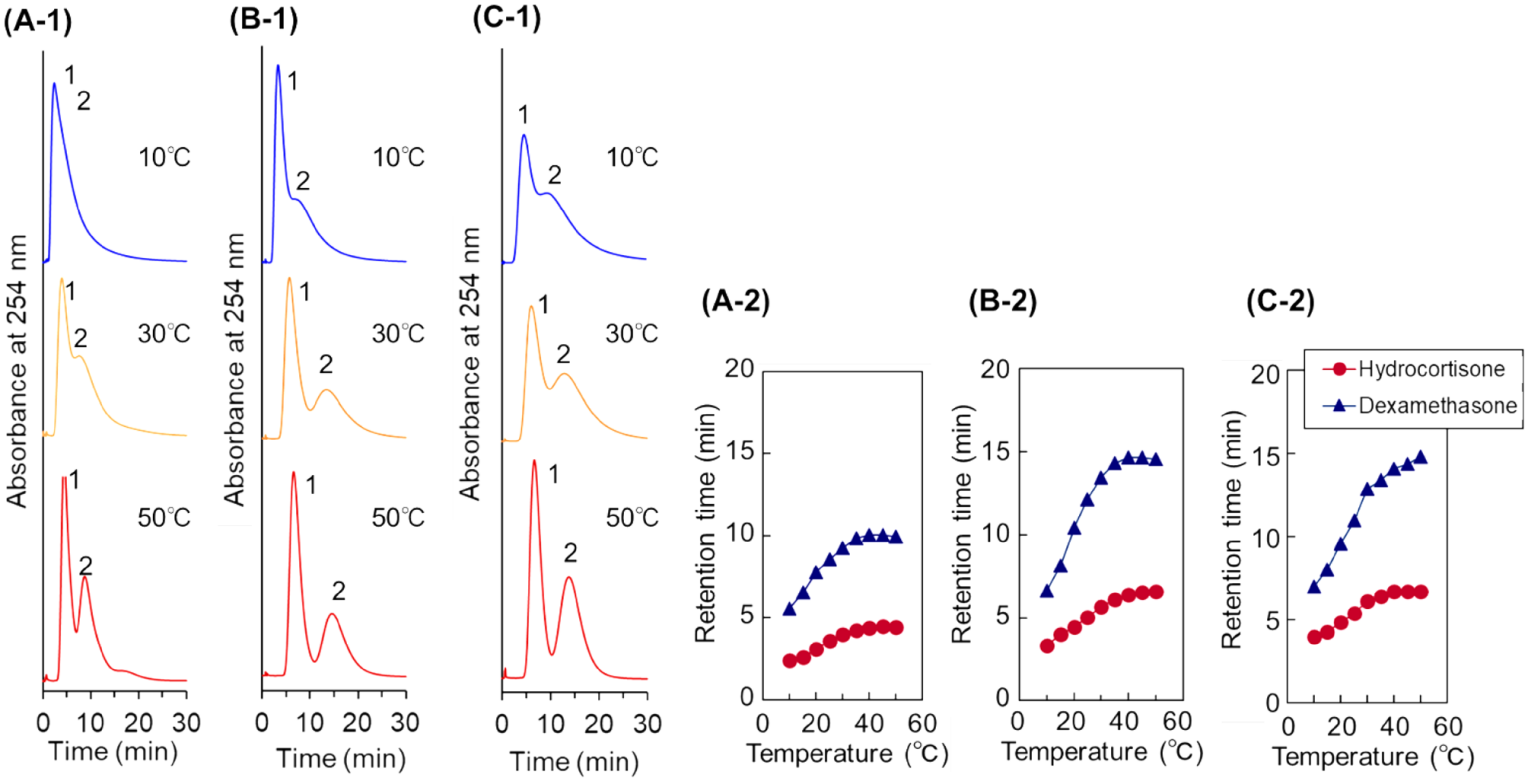
Chromatograms of steroids using temperature-responsive polymer modified beads columns (− 1) and retention time of steroids at various temperatures (− 2). (**A**) P(NIPAAm-*co*-AMPS-*co*-PhAAm)-modified beads column, (**B**) P(NIPAAm-*co*-AMPS-*co*-BMA)-modified beads column, and (**C**) P(NIPAAm-*co*-AMPS-*co*-tBAAm)-modified beads column. The mobile phase is a 33.3 mM phosphate buffer solution with a flow rate of 1.0 mL/min. Peak 1 represents hydrocortisone; 2, dexamethasone.

The prepared polymer-modified beads were packed into a stainless steel column, and the packed column was connected to a high performance liquid chromatography (HPLC) system. To investigate the hydrophobicity of the prepared beads, the elution behaviour of hydrophobic steroids from the columns was observed at various temperatures (Fig. 3). The properties of steroids are summarised in Supplementary Table S4. Two steroids, hydrocortisone and dexamethasone, were used as model hydrophobic analytes to investigate the hydrophobic interaction between analytes and thermoresponsive anionic polymers on the beads, because these steroids have a relatively strong hydrophobicity and interact with the thermoresponsive polymers through hydrophobic interactions^6^. In addition, these steroids do not electrostatically interact with thermoresponsive anionic polymers, because the steroids do not have acidic or basic groups^39,41^. Thus, the elution behaviour of these steroids predicted the hydrophobic interaction between analytes and thermoresponsive anionic polymers on the beads. In the chromatogram of the steroids from the columns, the steroids were eluted from the column in the order of the log*P*, hydrocortisone (log*P* = 1.61), and dexamethasone (log*P* = 1.83). This indicated that the retention of steroids on the column resulted from a hydrophobic interaction. The retention time increased with increasing the column temperature, and separation was performed at higher temperatures. This was because the thermoresponsive anionic polymers on the beads became dehydrated and interacted with the steroids through a hydrophobic interaction as the temperature was increased. Among the three types of beads, the P(NIPAAm-*co*-AMPS-*co*-BMA) beads-packed column exhibited the largest retention time and relatively good separation compared with the other columns. This indicated that the P(NIPAAm-*co*-AMPS-*co*-BMA) beads exhibited a relatively strong hydrophobicity compared with those columns packed with the other polymers. BMA exhibited the greatest hydrophobicity (log*P* = 2.23) compared with PhAAm (log*P* = 1.63) and tBAAm (log*P* = 0.84). Thus, the P(NIPAAm-*co*-AMPS-*co*-BMA) beads-packed column exhibited longer retention time and better separation of the steroids mixture compared with those obtained with the P(NIPAAm-*co*-AMPS-*co*-PhAAm)- and P(NIPAAm-*co*-AMPS-*co*-tBAAm)-modified beads, because of the strong hydrophobicity of BMA and the hydrophobic interaction with analytes. In the comparison of the P(NIPAAm-*co*-AMPS-*co*-BMA) beads with the P(NIPAAm-*co*-AMPS-*co*-PhAAm) beads, P(NIPAAm-*co*-AMPS-*co*-BMA) contains a larger hydrophobic monomer unit, BMA 5 mol%, compared with PhAAm 1 mol% of P(NIPAAm-*co*-AMPS-*co*-PhAAm). Also, BMA has higher hydrophobicity compared with PhAAm. Thus, P(NIPAAm-*co*-AMPS-*co*-BMA) has a higher hydrophobicity compared with P(NIPAAm-*co*-AMPS-*co*-PhAAm). In contrast, by comparison of P(NIPAAm-*co*-AMPS-*co*-BMA) and P(NIPAAm-*co*-AMPS-*co*-tBAAm), P(NIPAAm-*co*-AMPS-*co*-tBAAm) has quite a large hydrophobic monomer composition, 20 mol% of tBAAm, compared with P(NIPAAm-*co*-AMPS-*co*-BMA), 5 mol% of BMA. However, P(NIPAAm-*co*-AMPS-*co*-BMA)-modified beads exhibited a strong retention of hydrophobic steroids. This was proposed to arise from the difference in the basic monomer structures between BMA and tBAAm. BMA and tBAAm have a methacrylate and acrylamide monomer structure, respectively. The methacrylate monomer has a more hydrophobic structure compared with acrylamide. Thus, P(NIPAAm-*co*-AMPS-*co*-BMA) exhibited a strong hydrophobic interaction with steroids, which led to a longer retention time.

**Figure 4:**
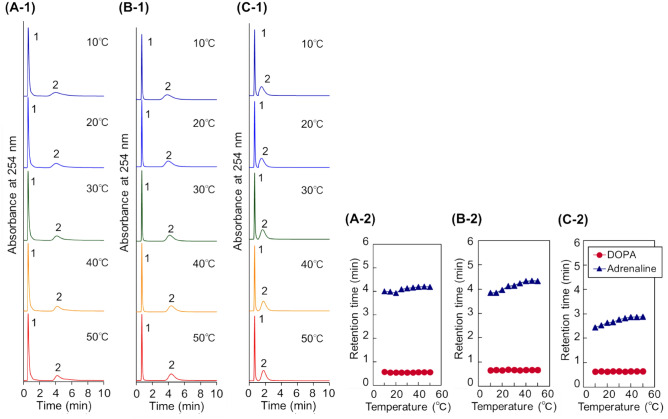
Chromatograms of catecholamines using temperature-responsive polymer modified beads-packed columns (− 1) and retention time of catecholamines at various temperatures (− 2). (**A**) P(NIPAAm-*co*-AMPS-*co*-PhAAm)-modified beads-packed column, (**B**) P(NIPAAm-*co*-AMPS-*co*-BMA)-modified beads-packed column, and (**C**) P(NIPAAm-*co*-AMPS-*co*-tBAAm)-modified beads-packed column. The mobile phase is a 33.3 mM phosphate buffer solution at pH 7.0 with a flow rate of 1.0 mL/min. Peak 1 represents DOPA; 2, Adrenaline.

Additionally, the anionic property of the prepared beads was investigated by observing the elution behaviour of catecholamines, 3-(3,4-dihydroxyphenyl)-_L_-alanine (DOPA) and adrenalin (Fig. 4). The properties of catecholamines are summarised in Supplementary Table S4. These analytes have a basic property (DOPA (p*K*a = 8.72) and adrenalin (p*K*a = 9.8–9.9)). Thus, the retention behaviour of these catecholamines predicted the electrostatic interaction between the basic analytes and the modified thermoresponsive anionic polymers on the beads^28^. The catecholamines eluted from the column in the order of the basicity, DOPA (p*K*a = 8.72) and adrenalin (p*K*a = 9.8–9.9). This indicated that these analytes were mainly retained through the electrostatic interaction between the sulfonic acid of AMPS and the analytes. Also, the two catecholamines were separated on all columns, which indicated that all columns exhibited a sufficient electrostatic interaction for the separation of the analytes. The retention time of adrenaline was slightly increased with an increase of the temperature. This was attributed to the increased anionic property with increasing temperature, as predicted from the zeta potential of the beads (Fig. 2C). Namely, the thermoresponsive anionic polymer on the beads shrunk with increasing temperature, which led to the increased apparent charge density on the beads at high temperature. Additionally, the hydrophobic interaction between adrenalin and the thermoresponsive anionic polymer contributed to the retention. In a previous report regarding the separation of catecholamines with a P(NIPAAm-*co*-acrylic acid (AAc)-*co*-tBAAm)-modified beads-packed column, the catecholamines were retained on the column through electrostatic and hydrophobic interactions, although the electrostatic interaction was the main driving force^37^. Thus, in the case of the columns used in this study, the catecholamines were also proposed to be retained on the column through electrostatic and hydrophobic interactions. Thus, the retention time of adrenaline increased with an increase of the temperature, because the modified polymers on the beads became hydrophobic upon increasing the temperature. Additionally, the increase in the retention time of adrenaline was most obvious with P(NIPAAm-*co*-AMPS-*co*-BMA). This was proposed to arise from the BMA monomer unit included in the polymer inducing a strong hydrophobic interaction with the analytes, the same as the retention of hydrophobic steroids.

### Antibody drug purification using columns

**Figure 5:**
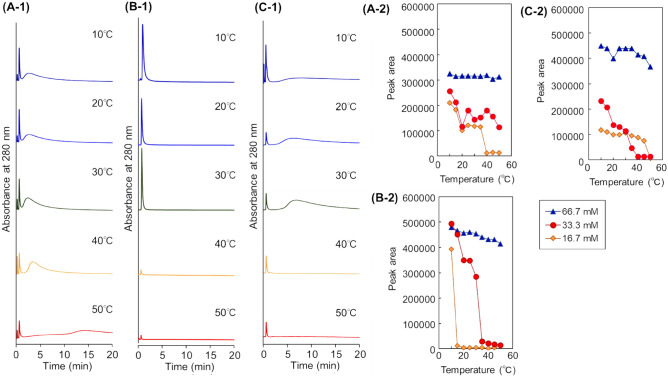
Elution behaviour of rituximab using temperature-responsive polymer modified beads-packed columns (− 1) and peak area of eluted rituximab at various temperatures (− 2). (**A**) P(NIPAAm-*co*-AMPS-*co*-PhAAm)-modified beads-packed column, (**B**) P(NIPAAm-*co*-AMPS-*co*-BMA)-modified beads-packed column, and (**C**) P(NIPAAm-*co*-AMPS-*co*-tBAAm)-modified beads-packed column. The mobile phase is a 33.3 mM phosphate buffer solution at pH 7.0 with a flow rate of 1.0 mL/min.

To investigate the availability of the prepared columns for application in biopharmaceuticals separation, the elution behaviour of an antibody drug on a column was observed using rituximab as a model antibody drug (Fig. 5). The properties of antibody drugs are summarised in Supplementary Table S5. Elution of rituximab was observed at the lower temperature region. However, by increasing the column temperature, the elution of rituximab was not observed. This was because of rituximab adsorption on the thermoresponsive anionic polymers at a high temperature. Rituximab has a weak positive charge in the neutral mobile phase^50^. Thus, rituximab was adsorbed through electrostatic interactions with the thermoresponsive anionic polymers on the beads in the column. Additionally, rituximab was adsorbed only at high temperature, which indicated that the hydrophobic interaction attributed to the dehydration of polymers at higher temperature also contributed to the adsorption of rituximab. Thus, rituximab was adsorbed on the thermoresponsive anionic polymer on the beads through both electrostatic and hydrophobic interactions. We investigated the effect of the ionic strength of the mobile phase on the adsorption of rituximab (Fig. 5), because the electrostatic interaction was the main driving force for the adsorption of rituximab. At a high concentration of the mobile phase, 66.7 mM, effective rituximab adsorption was not observed because of the insufficient electrostatic interaction between rituximab and the thermoresponsive anionic polymers on the beads. In contrast, at a low concentration of the mobile phase, 16.7 mM, effective rituximab elution was not observed, because of the excessive electrostatic interaction between rituximab and the thermoresponsive anionic polymers on the beads. These results indicated that temperature-modulated adsorption and desorption of rituximab could be performed by adjusting the mobile phase to a proper concentration.

Additionally, by comparing the three columns, the P(NIPAAm-*co*-AMPS-*co*-BMA)-modified bead-packed column exhibited a clear temperature-modulated adsorption and elution of rituximab. This was attributed to the effective hydrophobic interaction with the BMA-incorporated polymer similar to the retention of steroids and catecholamines. Thus, P(NIPAAm-*co*-AMPS-*co*-BMA)-modified beads are suitable for the retention of rituximab.

The composition of AMPS and BMA in the P(NIPAAm-*co*-AMPS-*co*-BMA) copolymer are important factors, because there is an optimal balance between the electrostatic and hydrophobic interactions for an effective interaction with an antibody that are attributed to AMPS and BMA, respectively. In a previous report, the optimal AMPS composition in a thermoresponsive polymer was investigated by changing the AMPS composition in the thermoresponsive polymer from 0 to 10 mol%^39^. As a result, 5 mol% of AMPS was a suitable composition for an effective interaction with proteins, because a higher AMPS composition above 5 mol% reduced the dehydration property of the thermoresponsive polymer with increasing temperature, which was attributed to the strong hydrophilic property of the copolymer^39^. Additionally, the optimal BMA composition in the thermoresponsive polymer for temperature-responsive chromatography was investigated in a previous study^42,51,52^. In these investigations, the retention of analytes increased with increasing the composition of BMA^42,51,52^. However, a BMA composition above 5 mol% reduced the hydration property of the thermoresponsive polymer, because of the excessive hydrophobicity of the incorporated BMA in the polymer^42^. These previous reports suggested that the composition of 5 mol% AMPS and 5 mol% BMA in the copolymer, which was used in this study, is suitable for the effective interaction with proteins.

**Figure 6:**
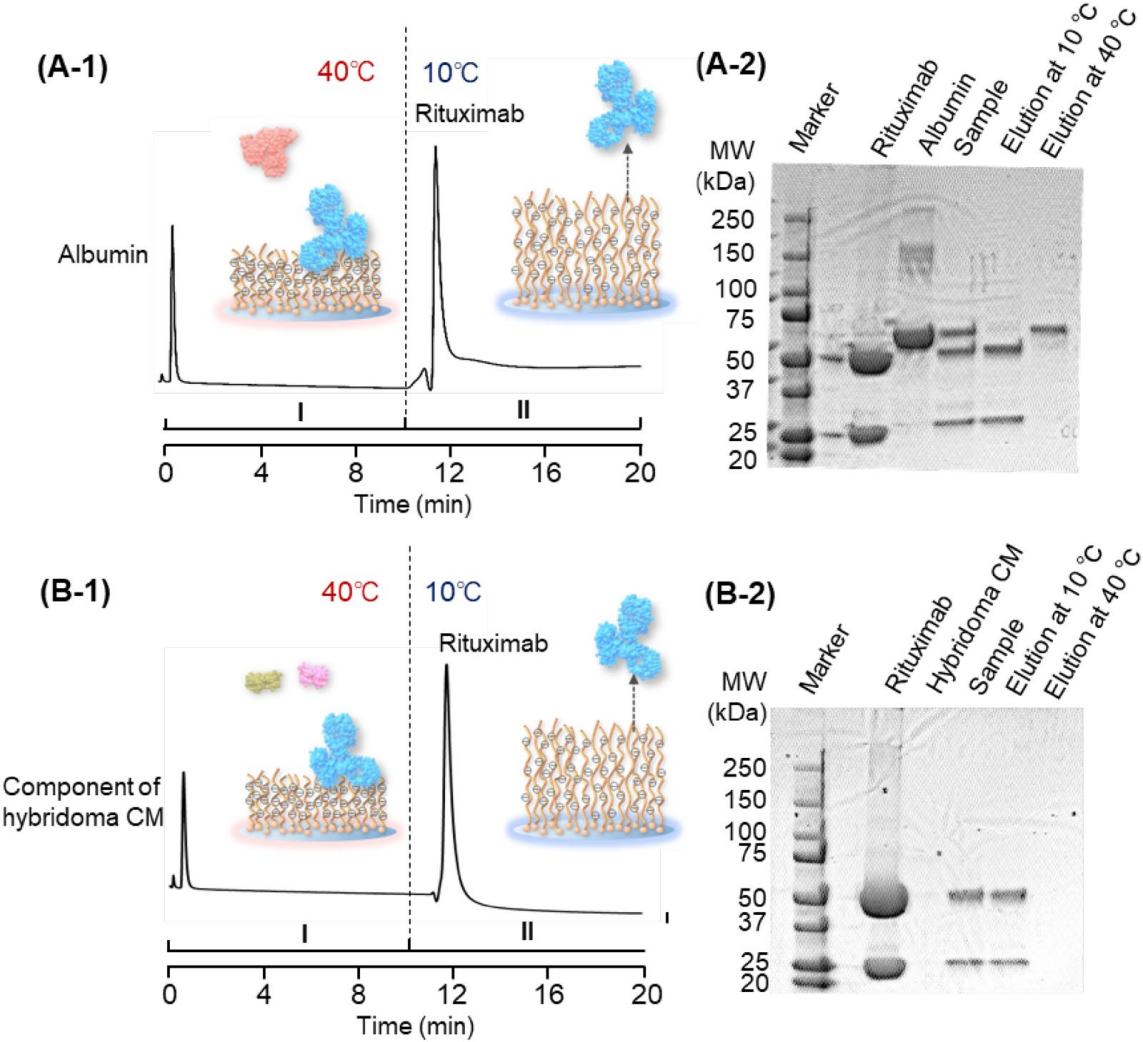
Purification of rituximab from protein mixtures using P(NIPAAm-*co*-AMPS-*co*-BMA)-modified beads-packed column by a step-temperature gradient. (**A**) Separation of a mixture of rituximab and albumin. (**B**) Purification of rituximab from hybridoma cell culture medium. Mobile phase is a 33.3 mM phosphate buffer solution.

Using the temperature-modulated rituximab adsorption property of the P(NIPAAm-*co*-AMPS-*co*-BMA)-modified beads-packed column, rituximab separation was performed by a step temperature gradient (Fig. 6). A mixture of rituximab and albumin, and a mixture of rituximab and hybridoma cell culture medium were used as model analytes, because albumin is the main contaminant in the antibody production process, and a hybrydoma cell culture medium is used for antibody production.

At 40 °C, the mixture of rituximab and albumin sample was injected into the column, and rituximab was adsorbed and albumin was eluted. Then, the column temperature was reduced to 10 °C, and the adsorbed rituximab was eluted. Sodium dodecyl sulfate polyacrylamide gel electrophoresis (SDS-PAGE) analysis indicated that the elution fractions at 40 °C and 10 °C contained albumin and rituximab, respectively. Two bands were observed in the lane of the rituximab, which arose from the degradation of rituximab into a light chain and heavy chain through the SDS-PAGE analysis. This indicated that the mixture sample of rituximab and albumin can be separated simply by changing the column temperature.

Furthermore, for the mixture of rituximab and hybridoma cell culture medium, the mixture sample was injected into the column at 40 °C, and rituximab was adsorbed and the component of the hydridoma cell culture medium was eluted. Then, the column-temperature was reduced to 10 °C, and the adsorbed rituximab was eluted from the column. SDS-PAGE analysis indicated that the fraction eluted at 40 °C did not contain rituximab and that at 10 °C contained rituximab. The results indicated that rituximab purification from the cell culture medium can be performed simply by changing the column temperature.

In these experiments, albumin and the component of hybrydoma cell culture medium were eluted at probably the same retention time, which indicates that albumin and the component of hybrydoma cell culture medium would not be separated. However, the separation of albumin and the component of hybrydoma cell culture medium is not necessary for the actual antibody purification process. In the production process, the antibody and albumin are dissolved in hybrydoma cell culture medium. In the purification process, the antibody should be separated from the albumin and the component of hybridoma cell culture medium. Thus, the separation of albumin and the component of hybrydoma cell culture medium is not important for the application of antibody purification.

The activity of the eluted rituximab from the column was evaluated by observing the reactivity with CD20-positive human B cell leukaemia cells, because rituximab is an anticancer drug that recognizes CD20-positive cells. The rituximab activity with B cell leukaemia cells was maintained after column elution compared with that before column injection (Supplementary Fig. S4). This result demonstrated that the purified rituximab, after passing through the column, maintained its drug activity.

**Figure 7:**
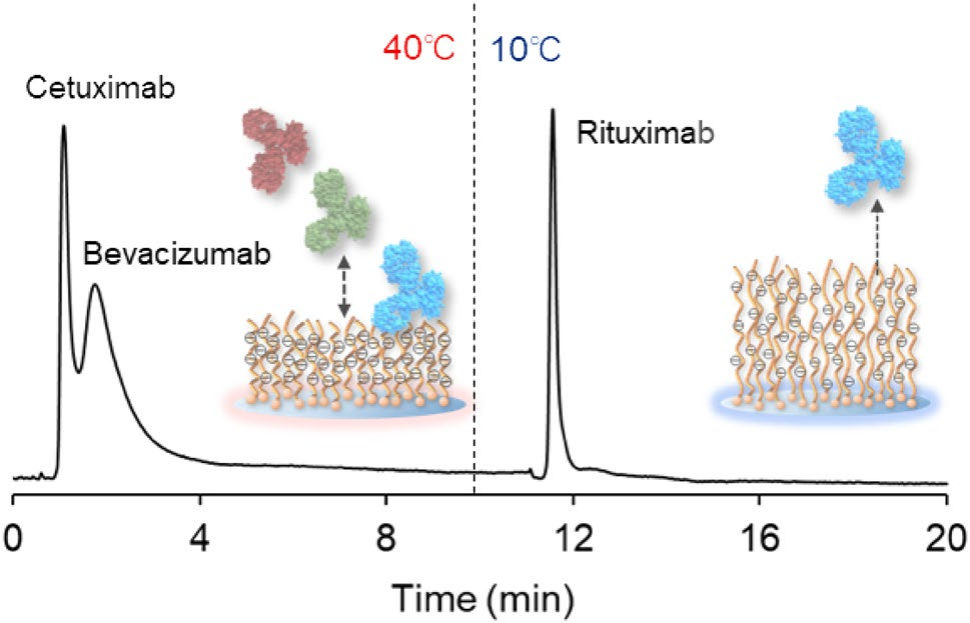
Separation of antibody drugs using P(NIPAAm-*co*-AMPS-*co*-BMA)-modified beads-packed column. Mobile phase is a 30.0 mM phosphate buffer solution at pH 6.0. Column length is 150 mm.

Finally, we investigated the column performance on the separation of an antibodies mixture where the antibodies have different structures. In the production of antibody drugs, small amounts of antibodies with slightly different structures contaminate the production. Thus, purification of the antibody from a mixture of various structures of antibodies would be useful in this situation. Thus, a mixture of three antibody drugs, composed of cetuximab, bevacizumab, and rituximab, was used as a model antibody mixture where the antibodies had slightly different structures, and separation of these three antibodies was performed using the column (Fig. 7 and Supplementary Fig. S5).

At 40 °C, the mixture of cetuximab, bevacizumab, and rituximab was injected into the column. Cetuximab and bevacizumab were eluted at 40 °C, and rituximab was adsorbed in the column. In the elution of cetuximab and bevacizumab, bevacizumab was slightly retained in the column compared with cetuximab, which was proposed to arise from the difference in electrostatic and hydrophobic properties between cetuximab and bevacizumab. This led to the separation of these antibody drugs. Then, the column temperature was reduced to 10 °C, and the adsorbed rituximab was eluted. The results demonstrated that the three types of antibody drugs can be separated by using the developed column. Ordinarily, antibody drugs can be purified using a protein A ligand column^5^. Protein A recognizes the Fc region of antibodies, which commonly exists in antibodies. Thus, a Protein A column has difficulty to separate a mixture of antibody drugs. In contrast, the developed temperature-responsive column can separate a mixture of three types of antibody drugs simply by changing the temperature. The column would be a useful tool for antibody separation and analysis.

The prepared thermoresponsive anionic polymer modified beads-packed column exhibited an effective interaction with small and large molecular analytes by incorporating BMA as the hydrophobic monomer. The column can perform temperature-dependent antibody drug adsorption and desorption, and can purify an antibody drug from a mixture of contaminants simply by changing the temperature. Thus, the column would be useful for the purification and separation of antibody drugs.

## Conclusions

Three types of thermoresponsive anionic copolymer brush modified beads were developed as packing materials for functional temperature-responsive chromatography columns for functional antibody drug separation. To develop the thermoresponsive anionic copolymer brush modified beads, AMPS was used as a strong anionic monomer, and the hydrophobic monomers, PhAAm, BMA, and tBAAm, were used as a hydrophobic monomer. The electrostatic and hydrophobic properties of the prepared beads were investigated by observing the elution behaviour of hydrophobic steroids and catecholamines from the beads-packed column. Among the three types of columns, the P(NIPAAm-*co*-AMPS-*co*-BMA) modified beads-packed column exhibited an effective retention of analytes, which was attributed to the strong hydrophobic interaction. To investigate the suitability of the prepared column to a biopharmaceuticals application, the elution behaviour of rituximab was observed. Rituximab was adsorbed on the column at high temperature, while rituximab was eluted from the column at low temperature. The P(NIPAAm-*co*-AMPS-*co*-BMA) modified beads-packed column exhibited the most effective adsorption and elution of rituximab among the three types of columns. Using the temperature-dependent adsorption and desorption properties, rituximab was purified from contaminants. Furthermore, three types of antibody drugs were separated using the column simply by changing the temperature. These results demonstrate that the prepared thermoresponsive anionic copolymer modified beads-packed column will be useful for bioseparation and bioanalysis for biopharmaceutics simply by changing the column temperature.

## Methods

### Preparation of thermoresponsive anionic polymer grafted beads

Silica beads grafted with a thermoresponsive anionic polymer brush were prepared by the following procedure (Fig. 1). Silica beads (10 g) were washed with hydrochloric acid for 2 h at 90 °C. Then, the beads were rinsed with a large amount of water and dried for 8 h at 150 °C. The beads were reacted with CPTMS through silanization, which acted as the initiator of ATRP. The CPTMS reaction solution was prepared by dissolving 2.7 mL of CPTMS in 200 mL of toluene. The solution was reacted with the beads for 18 h at 25 °C. After the reaction, the silica beads were rinsed with toluene and acetone, and dried for 3 h at 110 °C.

The polymer-modified beads were prepared by ATRP of the thermoresponsive monomer, anionic monomer, and three types of hydrophobic monomers. The anionic monomer AMPS (3.11 g, 15.0 mmol) was dissolved in 20 mL of water. Then, the AMPS solution was neutralised by adding NaOH aqueous solution, and the total volume was adjusted to 50 mL. To prepare the monomer solution containing PhAAm as a hydrophobic monomer, NIPAAm (1.37 g, 12.1 mmol) and PhAAm (18.9 mg, 0.12 mmol) were dissolved in 2-propanol (38.5 mL) in a flask. The prepared AMPS solution (2.14 mL) and water (2.14 mL) were added to the solution. To prepare the BMA-containing monomer solution, NIPAAm (1.31 g, 11.6 mmol) and BMA (91.6 mg, 0.64 mmol) were used. To prepare the tBAAm-containing monomer solution, NIPAAm (1.10 g, 9.67 mmol) and tBAAm (0.328 g, 2.58 mmol) were used. The prepared monomer solutions were deoxygenated by flowing argon for 15 min. Then, CuCl (84.7 mg, 0.86 mmol), CuCl_2_ (11.5 mg, 0.086 mmol), and Me_6_TREN (221 mg, 0.959 mmol) were added into the solution with continuous argon gas bubbling. The flask was sealed after filling with argon gas. The CPTMS beads (1.0 g) were put into a glass vessel. The ATRP reaction solution in a flask and the CPTMS beads in a glass vessel were placed into a glove bag. Oxygen in the glove bag was removed by a cycle of vacuuming and flowing argon. The ATRP reaction solution was added to the CPTMS beads in the glass vessel. The glass vessel was sealed. The ATRP reaction proceeded with shaking of the glass vessel for 16 h at 25 °C. After the reaction, the beads were washed with acetone, EDTA aqueous solution, methanol, and water, followed by drying in reduced pressure.

To characterise the thermoresponsive polymer, an unbounded polymer was synthesised using the same procedure except that α-chloro-*p*-xylene (1.75 μL, 13.3 μmol) was used in place of the CPTMS-modified silica beads. The synthesised polymer was purified through dialysis using a cellulose dialysis tube (1 kDa MWCO).

### Characterisation of thermoresponsive anionic polymer grafted beads

The prepared polymer-modified silica beads were characterised by CHN elemental analysis, FTIR, scanning electron microscopy (SEM) observation, zeta potential observation, and observation of the transmittance change of the polymer solution.

The amount of ATRP-initiator and polymers on the silica beads was estimated by the carbon content obtained from CHN elemental analysis (PE2400, PerkinElmer, Waltham, MA, USA). The immobilised initiator was obtained from the equation:

1 $$\frac{{\% C_{I} }}{{\% C_{I} (calcd) \times \left( {1 - \% C_{I} /\% C_{I} (calcd)} \right) \times S}}$$

where %*C*_*I*_ is the increase in the percentage of carbon in the ATRP-initiator modified beads compared with the unmodified beads following a silane coupling reaction; %*C*_*I*_ (*calcd*) is the calculated percentage of carbon in the initiator, and *S* is the surface area of the beads (100 m^2^ g^−1^). The amount of polymer on the silica beads was obtained using the equation:

2 $$\frac{{\% C_{P} }}{{\% C_{P} (calcd) \times \left( {1 - \% C_{P} /\% C_{P} (calcd) - \% C_{I} /\% C_{I} (calcd)} \right) \times S}}$$

where %*C*_*p*_ is the increase in the percentage of carbon in the polymer-modified beads compared with the initiator-modified beads. %*C*_*P*_ (*calcd*) is the calculated percentage of carbon in the initiator.

The morphology of the beads at each reaction step was observed by FE-SEM (S-4700 microscope, Hitachi High Technologies, Tokyo, Japan).

Polymer modification onto the silica beads was confirmed using an FTIR spectrometer (FTIR-4700; JASCO, Tokyo, Japan).

Surface area and pore diameter of the beads were measured by nitrogen adsorption using a surface area analyser (Belsorp-maxII, MicrotacBEL, Osaka, Japan).

The zeta potential and diameter of the polymer-modified beads were measured using a zeta potential analyser (Zetasizer Nano-ZS, Malvern Instruments, Malvern, UK). The polymer-modified beads were suspended in a 5 mM potassium chloride solution. The zeta potential of the beads was observed from 10 to 50 °C.

The polymer phase transition was observed by the temperature-dependent optical transmittance change of the polymer solution. A polymer solution (0.5 wt%/v) was prepared at pH 7.0, using a 33.3 mM phosphate buffer solution.

### Elution behaviour of analytes from beads-packed columns

Prepared beads were packed into stainless steel columns (4.6 mm in diameter and 50 mm in length). For three types of antibody separation, a column that was 4.6 mm in diameter and 150 mm in length was used. Prepared beads were dispersed into a water and methanol (1:1) mixture solution. The dispersion was added into a column packer connected to a column. Packing of the beads was performed by flowing a water and methanol (1:1) mixture solution into the packer at a constant pressure of 350 kg cm^−2^ for 1 h. The elution behaviour of the analyte from the beads-packed column was observed on an HPLC system (Prominence-i LC-2030C, Shimadzu, Kyoto, Japan). The properties of the analytes are summarised in Supplementary Tables S1 and S2. All samples were prepared at a concentration of 500 ppm using the mobile phase. Phosphate buffer solution (pH 7.0, 33.3 mM) was used as a mobile phase with a flow rate of 1.0 mL min^−1^. Elution of steroids and catecholamines was detected at 254 nm. Elution of rituximab was detected at 280 nm. Column temperature was controlled by an equipped column oven in the HPLC system. The eluted protein samples at each fraction were analysed by SDS-PAGE.

The eluted rituximab activity was evaluated by flow cytometry. Rituximab eluted from the column was incubated with CD20-positive BALL-1 cells. Then, PE mouse anti-human IgG was reacted with the rituximab on CD20-positive cells as a secondary antibody. The amount of CD20-positive cells with PE was evaluated by a flow cytometer (LSR II, BD, Franklin Lakes, NJ, USA).

## Supplementary information

Supplementary Information.
