# Supplementary material for: Antibody drug separation using thermoresponsive anionic polymer brush modified beads with optimised electrostatic and hydrophobic interactions
Source: Sci Rep. 2020 Jul 27;10:11896. doi: 10.1038/s41598-020-68707-7 (PMC7385495; doi:10.1038/s41598-020-68707-7)
Supplement: Supplementary file 1 — Supplementary Information. [file 41598_2020_68707_MOESM1_ESM.docx]

Supplementary Information

**Antibody Drug Separation using Thermoresponsive Anionic Polymer Brush Modified Beads with Optimised Electrostatic and Hydrophobic Interactions**

Kenichi Nagase*^1^, Saki Ishii^1^, Koji Ikeda^1^, Sota Yamada^1^, Daiju Ichikawa^1^, Aya Mizutani Akimoto^2^, Yutaka Hattori^1^, Hideko Kanazawa^1^

1. Faculty of Pharmacy, Keio University, 1-5-30 Shibakoen, Minato, Tokyo 105-8512, Japan.
2. Department of Materials Engineering, School of Engineering, The University of Tokyo, 7-3-1 Hongo, Bunkyo, Tokyo 113-8656, Japan

*Corresponding author: (Phone) +81-3-5400-1378; (E-mail) nagase-kn@pha.keio.ac.jp

**Materials**

*N*-Isopropylacrylamide (NIPAAm) was kindly provided by KJ Chemicals (Tokyo, Japan) and was purified by recrystallisation from *n*-hexane. *N*-phenyl acrylamide (PhAAm) was purchased from Sigma-Aldrich (St Louis, MO, USA). 2-Acrylamido-2-methylpropanesulfonic acid (AMPS), 3-(3,4-dihydroxyphenyl)-L-alanine (DOPA), and adrenalin were purchased from Tokyo Chemical Industry (Tokyo, Japan). *n*-Butyl methacrylate (BMA), *tert*-butylacrylamide(tBAAm), tris(2-aminoethyl)amine (TREN), CuCl, CuCl_2_, toluene, 2-propanol, hydrocortisone, dexamethasone, and bovine serum albumin (BSA) were purchased from Fujifilm Wako Pure Chemical (Osaka, Japan). BMA was purified by distillation. Me_6_TREN was synthesised using TREN through the previously-reported procedure [^1^](#_ENREF_1). [(Chloromethyl)phenylethyl] trimethoxysilane (CPTMS) was purchased from Gelest (Morrisville, PA, USA). Rituximab was obtained from Zenyaku Kogyo (Tokyo, Japan). Hybridoma cell culture medium (Hybridoma-SFM) was obtained from Thermo Scientific (Rockford, IL, USA). Silica beads (diameter: 5 μm; pore size: 300 Å; and specific surface area: 100 m^2^ g^−1^) were obtained from Macherey-Nagel (Düren, Germany). Stainless steel columns (inner diameter: 50 × 4.6 mm) were purchased from GL Science (Tokyo, Japan).

**Supplementary Table S1.** Properties of hydrophobic monomer

| Monomers | Structure | Molecular weight | log*P* ^a^ |
| --- | --- | --- | --- |
| *N*-phenylacrylamide (PhAAm) |  | 147.18 | 1.63 |
| *n*-butyl methacrylate (BMA) |  | 142.20 | 2.23 |
| *tert*-butylacrylamide (tBAAm) |  | 127.19 | 0.84 |

a) Calculated by using Crippen’s fragmentation.[^2^](#_ENREF_2)


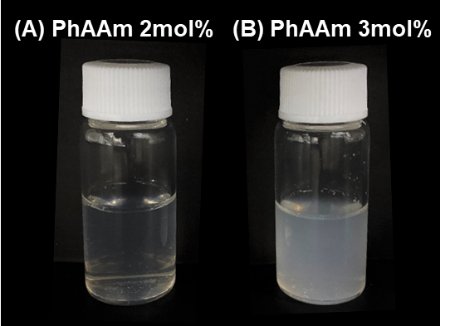


**Supplementary Figure S1.** Transparency of the solution prepared by dissolving the P(NIPAAm-*co*-AMPS-*co*-PhAAm) polymer in phosphate buffer using P(NIPAAm-*co*-AMPS-*co*-PhAAm) (A) containing 2 mol% PhAAm, and (B) containing 3 mol% PhAAm.

| Polymers | *M_n_* ^a^ | *M_w_* ^a^ | *M_w_*/*M_n_* ^a^ | LCST (°C) ^b^ |
| --- | --- | --- | --- | --- |
| P(NIPAAm-*co*-AMPS-*co*-PhAAm) | 7000 | 8300 | 1.18 | 35.6 |
| P(NIPAAm-*co*-AMPS-*co*-BMA) | 5800 | 6600 | 1.14 | 33.1 |
| P(NIPAAm-*co*-AMPS-*co*-tBAAm) | 5600 | 7700 | 1.37 | 21.9 |

**Supplementary Table S2.** Property of the thermoresponsive-anionic polymers

a) Determined by GPC using DMF containing 20 mM LiCl. b) Defined as the temperature at which the optical transmittance of the polymer solution was 50%.


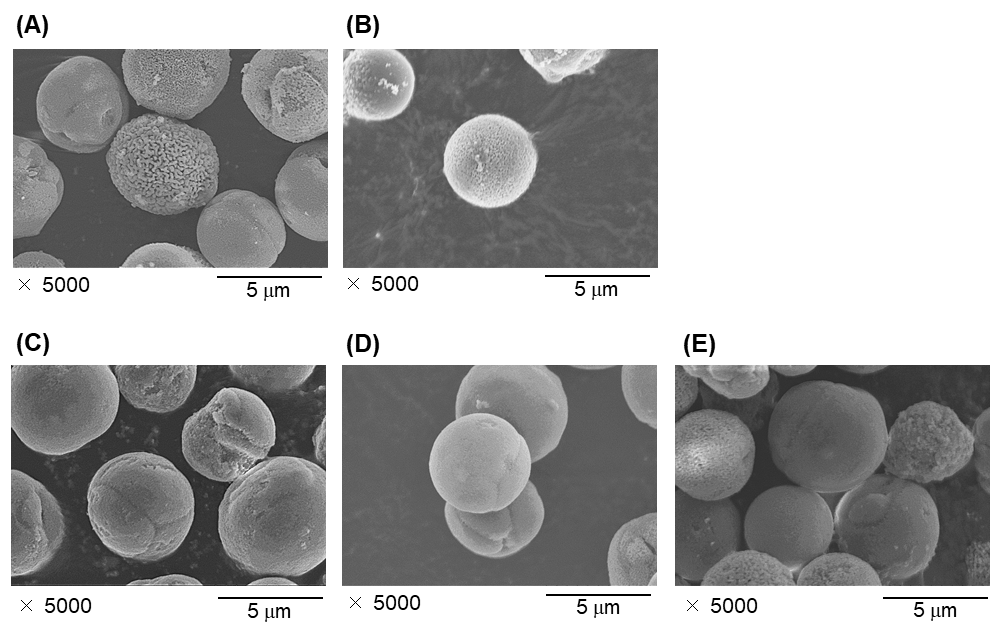


**Supplementary Figure S2.** Field-emission scanning electron microscopy (FE-SEM) images of (A) unmodified beads, (B) initiator-modified beads, (C) P(NIPAAm-*co*-AMPS-*co*-PhAAm)-modified beads, (D) P(NIPAAm-*co*-AMPS-*co*-BMA)-modified beads, and (E) P(NIPAAm-*co*-AMPS-*co*-tBAAm)-modified beads.

| Sample | Surface area ^a)^  (m^2^/g) | Total pore volume ^a)^  (cm^3^/g) | Peak pore diameter ^a)^  (nm) |
| --- | --- | --- | --- |
|  |  |  |  |
| P(NIPAAm-*co*-AMPS-*co*-PhAAm)-modified beads | 39.5 | 0.0166 | 1.68 |
| P(NIPAAm-*co*-AMPS-*co*-BMA)-modified beads | 59.7 | 0.0255 | 1.71 |
| P(NIPAAm-*co*-AMPS-*co*-tBAAm)-modified beads | 81.9 | 0.0332 | 1.62 |

**Supplementary Table S3.** Property of the thermoresponsive-anionic polymer modified beads measured by using nitrogen adsorption

a) Calculated using Brunauer-Emmett-Teller (BET) method.

**
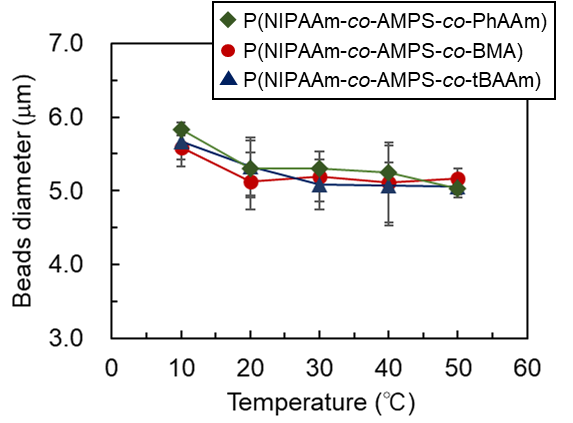
**

**Supplementary Figure S3** Diameter change of the temperature-dependent beads.

**Supplementary Table S4.** Properties of hydrophobic steroids and catecholamines

| Compounds | Structure | Molecular weight | Log*P* ^a^ | p*K* |
| --- | --- | --- | --- | --- |
| Hydrocortisone | 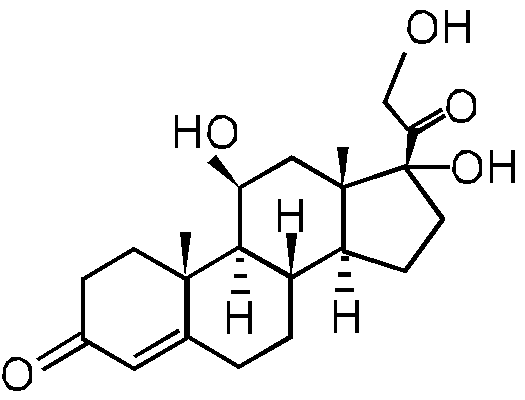 | 362.46 | 1.61 |  |
| Dexamethasone | 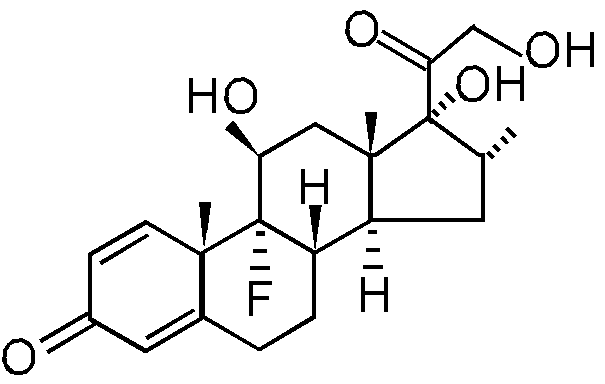 | 392.46 | 1.83 |  |
| DOPA | 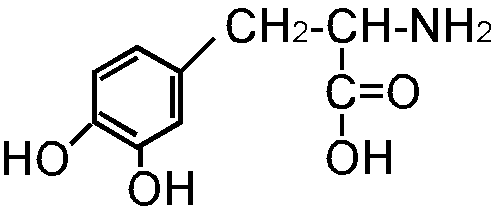 | 197.2 | −2.74 | 8.72 |
| Adrenaline | 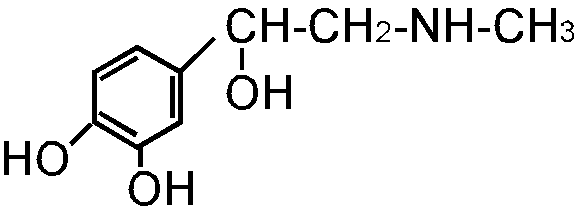 | 183.2 | −0.685 | 9.8–9.9 |

a) Partition coefficient in an *n*-octanol/water system.[^2^](#_ENREF_2)

**Supplementary Table S5** Properties of antibody drugs

| Analyte | Molecular weight (kDa) | p*I* | Remarks |
| --- | --- | --- | --- |
| Rituximab | 144 | 8.86 [^3^](#_ENREF_3) | Targets CD20-positive lymphoma |
| Cetuximab | 146 | 8.48 [^4^](#_ENREF_4) | Inhibits the epidermal growth factor receptor (EGFR) |
| Bevacizumab | 149 | 8.4 [^5^](#_ENREF_5)^,^[^6^](#_ENREF_6) | Inhibits vascular endothelial growth factor (VEGF) |


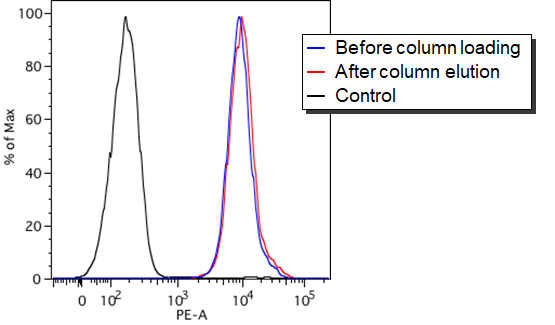


**Supplementary Figure S4.** Flow cytometry analysis of stained CD20-positive cells with rituximab and PE mouse anti-human IgG for evaluating activity of rituximab before and after column separation.


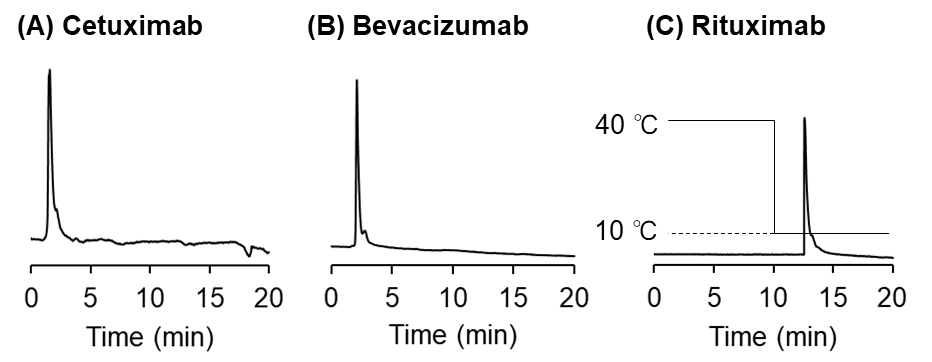


**Supplementary Figure S5.** Elution behaviour of antibody drugs from P(NIPAAm-*co*-AMPS-*co*-BMA)-modified beads-packed column for the peak identification of the chromatogram of the mixture sample.

**References**

1 Ciampolini, M. & Nardi, N. Five-Coordinated High-Spin Complexes of Bivalent Cobalt, Nickel, andCopper with Tris(2-dimethylaminoethyl)amine. *Inorg. Chem.* **5**, 41-44, doi:10.1021/ic50035a010 (1966).

2 Hansch, C., Albert, L. & Hoekman, D. in *Exploring QSAR: Hydrophobic, Electronic and Steric Constant ACS Professional Reference Book* (American Chemical Society, 1995).

3 Koetting, M. C., Guido, J. F., Gupta, M., Zhang, A. & Peppas, N. A. pH-responsive and enzymatically-responsive hydrogel microparticles for the oral delivery of therapeutic proteins: Effects of protein size, crosslinking density, and hydrogel degradation on protein delivery. *J. Control. Release* **221**, 18-25, doi:10.1016/j.jconrel.2015.11.023 (2016).

4 Kutty, R. V. & Feng, S.-S. Cetuximab conjugated vitamin E TPGS micelles for targeted delivery of docetaxel for treatment of triple negative breast cancers. *Biomaterials* **34**, 10160-10171, doi:10.1016/j.biomaterials.2013.09.043 (2013).

5 Vlčková, M., Kalman, F. & Schwarz, M. A. Pharmaceutical applications of isoelectric focusing on microchip with imaged UV detection. *J. Chromatogr. A* **1181**, 145-152, doi:10.1016/j.chroma.2007.12.046 (2008).

6 Nomoto, H. *et al.* Pharmacokinetics of Bevacizumab after Topical, Subconjunctival, and Intravitreal Administration in Rabbits. *Invest. Ophthalmol. Vis. Sci.* **50**, 4807-4813, doi:10.1167/iovs.08-3148 (2009).
